# Surveillance of Multidrug-Resistant Genes in Clinically Significant Gram-Negative Bacteria Isolated from Hospital Wastewater

**DOI:** 10.3390/antibiotics14060607

**Published:** 2025-06-15

**Authors:** Shriya C. Shetty, Lakshya S. Gowda, Ankeeta Menona Jacob, Kalidas Shetty, A. Veena Shetty

**Affiliations:** 1 Department of Microbiology, KS Hegde Medical Academy (KSHEMA), Nitte (Deemed to be University), Deralakatte, Mangalore 575018, India; n19phdm202@nitte.edu.in; 2 Department of Biosciences, Mangalore University, Mangalore 574199, India; lakshyagowda45@gmail.com; 3 Department of Community Medicine, KS Hegde Medical Academy (KSHEMA), Nitte (Deemed to be University), Mangalore 575018, India; dr.ankeetamj_kshema@nitte.edu.in; 4 Department of Microbiological Sciences, North Dakota State University, Fargo, ND 58102, USA; kalidas.shetty@ndsu.edu

**Keywords:** antimicrobial resistance, carbapenems, Gram-negative bacteria, treated effluent, wastewater

## Abstract

Background/Objectives: Antimicrobial resistance (AMR) has become a serious public health threat worldwide. Among the various surveillance domains, hospital wastewater (HWW) has been overlooked, and it is the major reason for the threats posed by AMR. Therefore, the HWW domain is of paramount importance for tackling the AMR. In this regard, the present study investigated the occurrence of Gram-negative bacteria from HWW and evaluated the isolates’ multi-drug-resistant (MDR) pattern in the study environment. Methods: This descriptive study involves HWW samples (*n* = 24) consecutively collected across 6 months. The samples were cultured for bacteria, identified, and subjected to antimicrobial susceptibility testing via Kirby–Bauer. PCR confirmed the presence of drug-resistance genes in Gram-negative bacterial isolates. Results: High rates of *Enterobacterales* resistant to carbapenems and cephalosporins observed in isolates from final treated effluent. The molecular screening showed *tet*D, *tet*E, *tet*G, *cat*A1, *cat*A2, *bla*_NDM-1_, quinolones, *qnr*A, *qnr*B, *qnr*S, and *qepa*. Conclusions: Overall, our results suggest that microbiological surveillance and identification of resistance genes of clinically important pathogens in HWW can be a general screening method for early determination of under-detected antimicrobial resistance profiles in hospitals and early warning of outbreaks and difficult-to-treat infections.

## 1. Introduction

Antibiotic resistance (AR) is emerging as one of the most critical global health concerns worldwide, with the potential to worsen significantly over the next decade due to demographic changes, environmental and healthcare system capacity, and socioeconomic and globalization. The World Health Organization (WHO) and the Joint Programme Initiative on Antimicrobial Resistance (JPIAMR) endorsed the role of the environment in selecting and disseminating the AR with higher priority for tackling the AR. Wastewater treatment is critical for environmental hygiene in urban environments. However, wastewater treatment plants (WWTPs) gather chemicals, organic materials, and microbes from many sources, including pathogens and multidrug-resistant (MDR) bacteria, which may be discharged into the environment through WWTP effluent. Although WWTPs are effective against enteric bacteria, they may also release other potentially harmful bacteria into the environment. WWTPs have recently been identified as hotspots for the enrichment, recombination, and selection of AR “super-bugs,” which could eventually be discharged and have a negative impact on the environment and human health, emphasizing the importance of water quality improvement strategies. The taxonomic resolution of full-length 16S rRNA genes enables more accurate characterization of possible pathogenic taxa and other microorganisms [1,2]. Antibiotic concentrations in wastewater and WWTPs, even those below the minimum inhibitory concentration (MIC), appear to be sufficient to promote the selection or transfer of resistance genes between bacteria. Additionally, antibiotic-resistant bacteria (ARB) that can colonize the human or animal gastrointestinal tract can reach the WWTPs by being eliminated through excreta [3]. The World Health Organization’s “One Health” strategy emphasizes the link between human, animal, and environmental health [4]. Accurate surveillance is essential for assessing the risk of AMR in a region, tracking the spread of antibiotic resistance genes (ARGs), identifying new ARGs, and implementing preventative measures against MDR pathogens [4]. Therefore, there is an urgent need to understand better the presence and distribution of antibiotic resistance genes in order to design effective treatment strategies and infection control measures. Resistance mechanisms involve various strategies, including upregulating efflux protein genes, downregulating influx protein genes, acquiring antibiotic-resistant genes (ARGs) by horizontal gene transfer from already resistant bacteria, and mutating antibiotic resistance genes. These resistance genes are usually encoded on plasmids or other mobile genetic components, enabling quick transfer within and across bacterial populations. Beta-lactam antibiotics are among the most frequently prescribed antimicrobials; yet, the rising incidence of beta-lactam resistance genes in bacterial populations reduces their efficacy. Beta-lactam resistance genes are more likely to occur and spread in bacterial populations as a result of their extensive usage, improper disposal of hospital waste, and indiscriminate use in livestock husbandry. Beta-lactam resistance is typically produced by enzymes that degrade the antibiotic’s beta-lactam ring, rendering it ineffective against bacteria. Genes for these enzymes, including *bla*_TEM_, *bla*_TEM_, *bla*_SHV1_, *bla*_CTX-M1_, *bla*_NDM_, and *bla*_VIM1,_ have been found in a number of Gram-negative bacterial species, with *Escherichia coli* having a significantly greater prevalence. Quinolone resistance, on the other hand, is caused by mutations in the *gyr*A, *gyr*B, *par*C, and *par*E genes, which reduce drug binding affinity. These genes are constitutively expressed and require further mutations in quinolone resistance-determining areas. Ciprofloxacin, levofloxacin, nalidixic acid, moxifloxacin, and other routinely prescribed quinolones lose efficacy when the *gyr*A, *gyr*B, *par*C, and *par*E genes are mutated, resulting in altered quinolone binding or lower affinity of protein products for quinolones. Critical missense mutations in specific areas of the gyrase and topoisomerase IV genes have a major impact on fluoroquinolone drugs’ capacity to create optimum configurations and bind to their target proteins. These areas, which include mis-sense mutations causing such effects, are called quinolone resistance-determining regions (QRDRs) [5]. Based on the background challenges of AMR, the overall goal of this study was to evaluate the antibiotic resistance profile of the prevailing Gram-negative bacteria in hospital wastewater effluent to determine whether they contribute to the spread of antibiotic resistance in the environment.

## 2. Results

### 2.1. Distribution of Gram-Negative Bacteria Across Hospital Wastewater Sites

Overall, 24 wastewater samples were collected and further investigated in the study. From this collection, 49 recovered antibiotic-resistant Gram-negative bacteria (AR-GNB) were isolated, comprising 33 from untreated wastewater and 16 from treated wastewater, dispersed over multiple sampling sites: Raw influent (SWW) 24.48% (*n* = 12), aeration tank (HWW-01) 24.48% (*n* = 12), recycled activated sludge (HWW-02) 18.36% (*n* = 9), and final effluent (HWW-03) 32.65% (*n* = 16) Table S1. Species identified were *Aeromonas* spp. 2.04% (*n* = 1), *Citrobacter freundii* 8.16% (*n* = 4), *Citrobacter koseri* 4.08% (*n* = 2), *E. coli* 26.53% (*n* = 13), *Enterobacter aerogenes* 2.04% (*n* = 1), *Klebsiella pneumoniae* 32.65% (*n* = 16), *Klebsiella oxytoca* 8.16% (*n* = 4), *Pseudomonas aeruginosa* 8.16% (*n* = 4), *Proteus mirabilis* 2.04% (*n* = 1), and *Proteus vulgaris* 6.12% (*n* = 3). Sampling carried out in the month of May 2023 showed the highest number of AR-GNB, 28.57% (*n* = 14), followed by April 2023, 26.53% (*n* = 13); December 2022, 22.44% (*n* = 11); February 2023, 18.36% (*n* = 9); and January 2023 and March 2023, 2.04% (*n* = 1).

### 2.2. Antibiotic Susceptibility Patterns of Gram-Negative Bacteria from Hospital Wastewater

All 49 Gram-negative bacteria recovered were found to be multidrug resistant. To determine resistance against antibiotic classes, the non-sensitivity of at least one of the antibiotics was considered for classes with multiple antibiotics. Aminoglycosides, Glycopeptides, and Sulfonamides showed a substantially better response in microbial suppression, with 12.5% *Citrobacter freundii* (*n* = 4), 50% *Citrobacter koseri* (*n* = 2), 38.46% *E*. *coli* (*n* = 13), 37.5% *Klebsiella pneumoniae* (*n* = 16), 50% *Klebsiella oxytoca* (*n* = 4), 16.6% *Proteus vulgaris* (*n* = 3), and 100% *Aeromonas* (*n* = 1), *Enterobacter aerogenes* (*n* = 1), and *Pseudomonas* (*n* = 4), indicating inefficiency to aminoglycosides. No resistance in *Aeromonas* (*n* = 1), *Citrobacter freundii* (*n* = 4), *Citrobacter koseri* (*n* = 2), *Klebsiella oxytoca* (*n* = 4), and *Enterobacter aerogenes* (*n* = 1); 38.46% *E coli* (*n* = 13), 25% of *Klebsiella pneumoniae* (*n* = 16), 75% of *Pseudomonas* (*n* = 4), and 100% of *Proteus vulgaris* (*n* = 3) and *Proteus mirabilis* (*n* = 1) showed inefficiency to sulfonamides. No resistance was shown in *Citrobacter koseri* (*n* = 2), *Enterobacter aerogenes* (*n* = 1), and *Proteus mirabilis* (*n* = 1), whereas 25% of *Citrobacter freundii* (*n* = 4), 23.07% *E coli* (*n* = 13), 31.25% *Klebsiella pneumoniae* (*n* = 16), 25% of *Klebsiella oxytoca* (*n* = 4), 50% *Pseudomonas* (*n* = 4), 33.3% *Proteus vulgaris* (*n* = 3), and 100% of *Aeromonas* (*n* = 1) indicated inefficiency to glycopeptides. While β-lactams, cephalosporins, ciprofloxacin, fluoroquinolones, carbapenems, quinolones, nitrofurantoin, and tetracyclines were found to be ineffective. Antimicrobial susceptibility testing revealed that all target AR-GNB isolates in this study were MDR.

### 2.3. MAR Indexing Pattern in Hospital Wastewater

The MAR index ranged from 0.3 to 0.7 in organisms and sampling sites between 0.48 and 0.54, indicating that the MAR values of all organisms and sites were from high-risk environments. (Table 1 and Table 2). *Aeromonas* spp. showed the highest MAR index (0.7), while *Enterobacter aerogenes* had the lowest (0.3). The sampling sites of the MAR index revealed that the untreated aeration tank (HWW-01) had the greatest value of 0.54, while treated final effluent (HWW-03) had the lowest value of about 0.48.

The ARI index indicated the prevalence of antibiotic-resistant and susceptible bacteria per organism and site. The present study observed from the ARI (Table 3 and Table 4) that beta-lactam and cephalosporin-resistant bacteria were more prevalent than those resistant to other classes of antibiotics in all sampling sites and organisms. Isolates obtained from raw influent (SWW) were found to have the highest resistance to β-lactams and cephalosporins. ARI distribution in organisms was different among sampling sites. *Citrobacter* spp. showed high resistance against phenicols and fluoroquinolones, while *E.coli* and *Klebsiella* spp. Showed resistance against β-lactams and *Proteus* spp. and *E.coli* against cephalosporins.

### 2.4. Screening for ARGs in WWTPs

**Figure 1:**
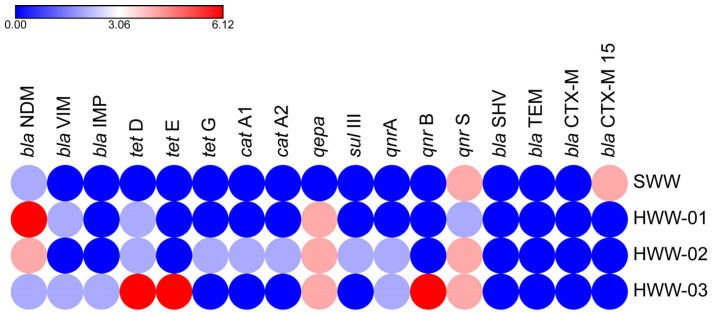
Occurrence of ARGs in sampling sites. Colored circles indicate the frequency of ARGs. Red circles denote the highest frequency, blue represents the lowest, and intermediate values are shown using a gradient between these two colors.

PCR confirmation of Gram-negative isolates in WTTPs identified 19 ARGs, 15 of which were expressed across all seven antibiotic classes. In the raw influent (SWW), *qnr*S was abundant, followed by *qnr*B, *bla*_NDM-1_, and *bla*_CTX-M15_, respectively. The aeration tank (HWW-01) contained carbapenems (*bla*_NDM-1_, *bla*_VIM_) and quinolones (*qnr*S, *qepa*), and in the recycled activated sludge (HWW-02), quinolones (*qnr*A, *qnr*S, *qepa*) conferred higher resistance followed by *bla*_NDM-1_, *tet*D, *tet*G, *sul*III, *cat*A1, and *cat* A2. Quinolones (*qnr*A, *qnr*B, *qnr*S, *qepa*), tetracycline (*tet*D, *tet*E), and carbapenems (*bla*_NDM-1_, *bla*_IMP_, *bla*_VIM_) were predominantly detected in the final effluent. None of the isolates was positive for *bla*_TEM_, *bla*_SHV_, *bla*_CTX-M_, *bla*_KPC_, and *bla*_OXA-48_ (Figure 1).

Among forty-nine Gram-negative isolates, nine of them harbored more than one ARG in the following combinations: *Aeromonas*: *cat*A1 + *cat*A2 + *qepa*, *C. freundii*: *tet*D + *tet*E, *E. coli*: *qnr*S + *bla*_CTX-M15_, *E.coli: qepa* + *bla*_NDM-1_, *E coli*: *tet*E + *qepa* + *qnr*B, *E.coli*: *qnr* B + *qnr* S, *Klebsiella pneumoniae*: *qnr*A + *bla*_NDM-1_, *Klebsiella oxytoca*: *tet*D + *tet* E + *qepa*, *Proteus vulgaris*: *tet* D + *qnr*B.

**Figure 2:**
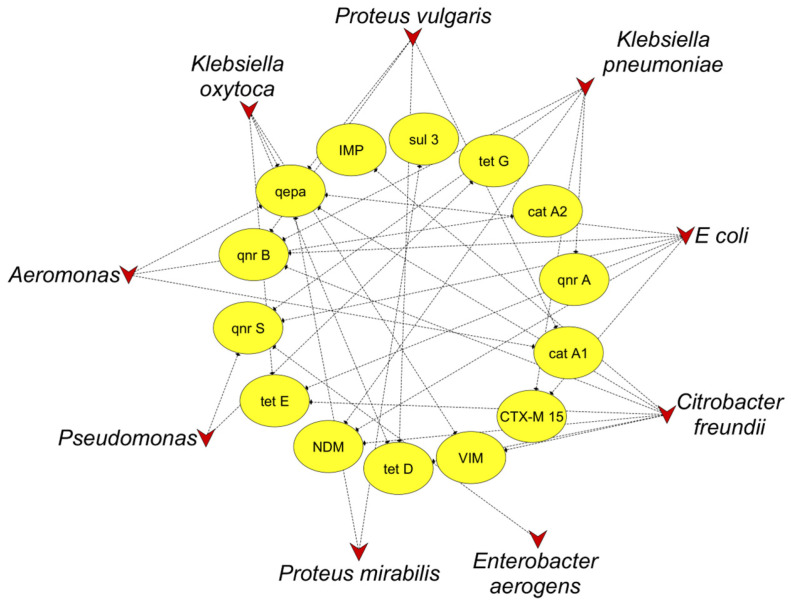
Co-occurrence network of ARGs among bacteria isolated from wastewater. The triangle and oval nodes indicate bacteria and targeted ARGs, respectively.

A network analysis was conducted using Cytoscape 3.9.1 to reveal the correlation between ARGs and the pathogenic bacterial community at the genus level in hospital wastewater. Our results showed that six genera, including *Aeromonas*, *Citrobacter*, *Escherichia*, *Klebsiella*, *Proteus*, and *Pseudomonas*, were positively correlated with at least two ARGs. Further, *Citrobacter*, *Escherichia*, and *Klebsiella* had clear correlations with numerous ARGs and were directly associated with genes that mediate resistance to carbapenems (*bla*_NDM-1_, *bla*_IMP_), tetracyclines (*tet*D, *tet*E), and quinolones (*qnr*A, *qnr*B, *qnr*S, *qepa*) (Figure 2). These findings suggest that *Enterobacterales* could be the primary host of ARGs and a potential MDR pathogen responsible for ARG dissemination into the environment via hospital wastewater.

## 3. Discussion

Unregulated wastewater discharge from hospitals and clinical sources into the environment is a significant public health concern in developing countries. This study assessed the presence of MDR bacteria in HWW at our facility. This study investigated the occurrence of various types of bacteria collected from different WWTP sites; HWW is a potential reservoir harboring drug-resistant, pathogenic, and opportunistic organisms. The transmission of ARB and their genes into the aquatic environment via the indiscriminate disposal of HWW is a severe concern [6]. Hospital wastewater surveillance is effective for monitoring antibiotic-resistant bacteria and the ARG load in the hospital setting. In reference to the Indian context, studies [4,7,8,9] have shown diversity and abundance of AR-GNB in hospitals posing risk to surrounding communities and the environment. In the current study, 33 AR-GNB were isolated from untreated wastewater and 16 AR-GNB from treated effluents, comprised primarily of *Aeromonas* spp., *Citrobacter* spp., *E. coli*, *Enterobacter aerogenes*, *Klebsiella* spp., *Pseudomonas aeruginosa*, and *Proteus* spp. Similar studies have identified bacterial species isolated from wastewater treatment plants [10,11,12]. Few studies additionally isolated *Acinetobacter* spp. and *Vibrio* spp. in their WTTPs [13,14,15,16], which were not present in our study. A prior meta-analysis found that bacteria isolated from HWW had an extensive range of resistance genes, including those encoding carbapenem, sulfonamide, tetracycline, and quinolone resistance [17]. The presence of such antibiotics in wastewater can exert selective pressure on the drug-resistant bacterial isolates. This investigation predominantly identified the MDR patterns against β-lactams, cephalosporins, ciprofloxacin, fluoroquinolones, carbapenems, quinolones, nitrofurantoin, and tetracyclines. The previous study [6] reported ESBL-producing *E. coli* were resistant to tetracycline, sulfonamide, ertapenem, and two third-generation cephalosporins: cefotaxime and cefpodoxime. Additionally, the isolates exhibited 75% resistance to ciprofloxacin, ceftazidime, and amoxicillin-clavulanate, and the MAR index ranged from 0.63 to 1.0. This is also evident in our study, as the MAR index ranged from 0.3 to 0.7 in organisms and sampling sites between 0.48 and 0.54, which is disturbing as final effluent (HWW-03) had a MAR index of 0.48, almost equivalent to raw influent (SWW) of 0.508. As suggested [13] in their study, the isolates might have originated from sources with a high contamination of antibiotics with MAR indices of the study sites (SWWTP, 0.35, and KWWTP, 0.33). Furthermore, the higher distribution of such MAR strains in HWW is due to Gram-negative enteric bacteria, which acquire several mechanisms against β-lactam antibiotics [18]. Other studies demonstrated the predominance of extended-spectrum beta-lactamases [5,6,10,19,20,21,22] in WWTPs; meanwhile, ARI indices show that beta-lactam and cephalosporin-resistant bacteria are more prevalent than other classes of antibiotics in all sample sites and organisms. The current study reveals that HWW is a significant reservoir for the persistence and dissemination of MDR bacteria, facilitating their escape from WWTPs into the environment. These findings are important from a public health perspective. HWW surveillance effectively tracks ARB and ARG levels in the hospital environment. The present study showed predominant carbapenems (*bla*_NDM-1_, *bla*_IMP_), tetracyclines (*tet*D, *tet*E), and quinolones (*qnr*A, *qnr*B, *qnr*S, *qepa*) genes, which were encountered in *Enterobacterales*, including *Citrobacter* spp., *E. coli*, *Enterobacter aerogenes*, *and Klebsiella* spp. isolated from wastewater samples. This result is in concordance with the high prevalence of carbapenems [12,14,22,23,24], tetracyclines [19], and quinolones [5,25,26,27,28] resistance-producing *Enterobacterales* [2], as HWW is considered to be a huge reservoir of ARB, and this could be linked to the volume and concentration of antibiotics present in the wastewater, which could have pre-disposed the bacteria therein to antibiotics. This could be responsible for the high resistance of bacteria in HWW to antibiotics. Several cephalosporin-resistant effluent isolates displayed resistance to 3rd generation cephalosporin antibiotics cefotaxime and ceftazidime, which was observed previously when the resistance patterns from [17,26,29] hospital waste were analyzed. Our previous report suggested the presence of the *bla*_NDM-1_ gene in untreated wastewater. While with treatment, treated water had no trace of ARGs [30]. A notable finding in this study is the presence of *tet*D, *tet*E, *qnr*A, *qnr*B, *qnr*S, *qepa*, *bla*_NDM-1_, *bla*_IMP_, and *bla*_VIM_ genes in treated final effluent (HWW-03). The resistance rates of fluoroquinolones are increasing, especially in *Enterobacterales*, due to their widespread use in treating both Gram-negative and Gram-positive bacterial infections. Of the known mechanisms of quinolone resistance, overexpression of efflux pumps and plasmid-mediated resistance, *qnr*B and S, which protect DNA gyrase from quinolone inhibition, were found in our strains. Proia et al. (2018) [31] and Azuma et al. (2019) [32] indicated that the discharge of untreated or insufficiently treated hospital effluent may facilitate the proliferation of ARB and ARGs. The presence of antibiotic residues increases selection pressure, leading to the acquisition of resistance among the aquatic environment’s indigenous microorganisms via transfer mechanisms such as conjugation, transformation, and transduction [25]. The horizontal gene transfer would be more preferred in such environments directly exposed to anthropogenic influences. Studies have shown persistent clonal lineages carry out exchange of plasmids encoding carbapenemase genes [12,33], mobile genetic elements [34], intergons, and plasmids [2], leading to horizontal gene transfer in the environment. This requires major resource allocation, adjustment of wastewater treatment techniques, and ongoing monitoring of AMR and antimicrobial groups. Indeed, HWW is now known to be a source of AMR transmission in the environment; hence, reducing AMR requires a One Health approach that takes into account all three compartments [35]. The present study also has some limitations. One among them was conducted at a single center. Also, the number of samples collected was relatively small and did not contain water samples from natural sources nearby, which might limit the interpretation of our conclusions. In addition, the sampling season may also have impacted bacterial isolation. Future studies with more focus on the HWW throughout the year with sampling at different intervals and expanding the sampling sites to nearby natural waters can give the precise knowledge about the transmission of MDR GNB isolates in this ecosystem chain. The affirmation of such elevated AR indicates the necessity to establish effluent standards tailored for healthcare facilities, while existing WWTPs should be enhanced with innovative treatment methodologies to address the reduction of emerging pollutants and the elimination of AR-GNB, thereby refining these strategies and fostering sustainable AMR mitigation in wastewater systems.

## 4. Materials and Methods

### 4.1. Sampling Sites and Collection of HWW

**Figure 3:**
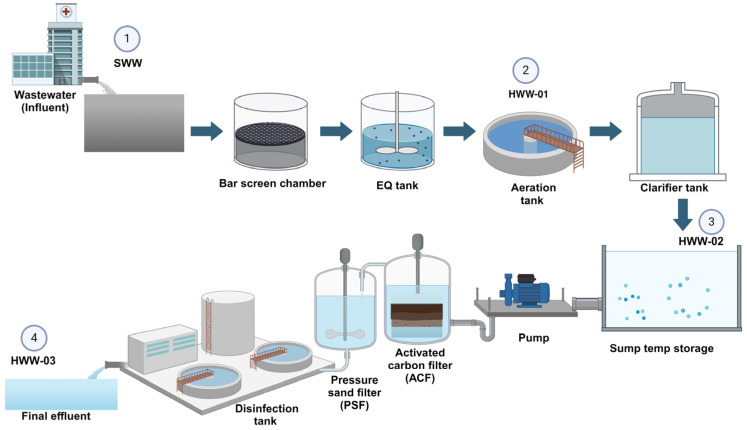
Schematic diagram of a hospital’s sewage treatment plant, with sampling sites as 1. Raw influent (SWW). 2. Aeration tank (HWW-01); 3. Recycled activated sludge (HWW-02) 4. Final effluent (HWW-03).

Samples of HWW effluents were collected once per month at four different sampling points from a tertiary care hospital (Mangalore, India) between December 2022 and May 2023. Two types of sampling points were considered: One from untreated wastewater (including raw influent (SWW), aeration tank (HWW-01), and recycled activated sludge (HWW-02) and another from treated wastewater, which included final effluent (HWW-03) (Figure 3).

Samples were collected from all four sites in 50 mL sterile tubes and transported to the laboratory immediately at room temperature for further analysis. A total of 18 untreated wastewater and 6 treated wastewater samples were collected over a period of 6 months.

### 4.2. Bacterial Isolation and Identification

After collection of wastewaters, to obtain isolated colonies, samples from wastewater effluents were serially diluted (ten-fold) with sterile physiological saline (0.85% NaCl), and 100 μL of each aliquot was spread onto non-selective Trypticase Soya Agar (TSA) plates followed by incubation at 37 °C for 24 h. From each plate, 10 morphologically distinct colonies were selected and subcultured on various selective media such as MacConkey agar, Eosin Methylene Blue (EMB) agar, Thiosulfate Citrate Bile sucrose Agar (TCBS), and Cetrimide Agar (HiMedia Laboratories Pvt. Ltd., Mumbai, India) for identification and further confirmed by a set of biochemical tests based on Bergey’s Manual [36].

### 4.3. Antibiotic Susceptibility Test

The Gram-negative bacterial isolates were tested for susceptibility against 20 antibiotics by the Kirby–Bauer method, using ATCC (USA) standard culture of *Escherichia coli* 25922 and *Pseudomonas aeruginosa* 27853 as quality control. Furthermore, the inhibition zones were measured and interpreted as sensitive or resistant as recommended based on results interpreted according to the M100 Performance Standards for Antimicrobial Susceptibility Testing, 32nd Edition [37]. Based on the guidelines, the isolates were categorized as MDR, extensive drug resistance (XDR), and pan-drug-resistant (PDR) when they showed resistance to >3, 14, and all 20 groups of antibiotics tested, respectively.

### 4.4. Determination of Antibiotic Resistance Pattern

The multiple antibiotic resistance (MAR) index was calculated as the ratio of total antibiotics used to antibiotics to which the bacterial isolate was resistant. A MAR index value higher than 0.2 indicates that the isolate is multiple antibiotic resistant [13,38]. The prevalence of antibiotic resistance based on the site of sample collection and organisms isolated is determined by the antibacterial resistance index (ARI) [38].

### 4.5. Bacterial DNA Extraction

Bacterial genomic DNA was extracted using a crude method. Briefly, 1 mL of 24 h grown culture in LB broth was taken in 1.5 mL microcentrifuge tubes, and the culture was centrifuged at 5000 rpm for 10 min. The supernatant was discarded, and the pellet was dissolved in 200 μL of 1X TE buffer. The tubes were placed in a dry bath at 95° C for 10 min and immediately placed in ice for another 10 min. After centrifugation at 5000 rpm for 5 min, the supernatant was transferred to a fresh microcentrifuge tube. The concentration and purity were determined in a spectrophotometer (Bio Spectrometer, Eppendorf, Hauppage, NY, Hamburg, Germany), and the crude DNA was stored at −20° C.

### 4.6. Detection of ARGs

The extracted DNA was analyzed by PCR for genes responsible for conferring resistance to different classes of antibiotics like tetracycline (*tet*D, *tet* E, *tet* G), sulfonamide (*sul*III), chloramphenicol (*cat*A1, *cat*A2), quinolones (*qnr*A, *qnr*B, *qnr*S, *qepa)*, β-lactams (*bla*_TEM_, *bla*_SHV_, *bla* _CTX-M_, *bla*_CTX-M15_), and carbapenems (*bla*_NDM-1_, *bla*_IMP_, *bla*_VIM_, *bla*_KPC_, *bla*_OXA-48_) using specific primers (Table 5). PCR was carried out in a 30 µL reaction containing 22.1 µL of sterile water, 3 µL of 10× assay buffer (100 mM Tris-HCL (pH 9), 1.5 mM MgCl2, 50 mM KCL, and 1% gelatin), 0.6 µL of 200 mM of four dNTP mix (dATP, dGTP, dCTP, and dTTP), 10 pmol of each forward and reverse primers, 1.0 U of Taq DNA polymerase, and 2 µL of template DNA in a thermal cycler (HiMedia, Mumbai, India) [39].

## 5. Conclusions

Overall, our findings suggest that HWW is a reservoir of MDR bacteria carrying carbapenemase, tetracycline, plasmid-mediated-quinolones, and quinolone-resistant genes categorized as a critical priority. This HWW ecosystem has the ability to encourage the spread of bacterial resistance, posing a risk for horizontal gene transfer, and this adds to the threat of AMR transmission via the environment. A timely evaluation of HWW is necessary, especially in hospitals with a high flow of hospitalizations, as well as changes in practice regarding the release of untreated effluents into the municipal sewage system. This study highlights the importance of wastewaters in One Health AMR surveillance programs to understand the emergence and transmission dynamics of AMR and for designing environmental intervention strategies.

## Figures and Tables

**Table 1:** MAR index value of Gram-negative bacteria.

Organism Identified	MAR Index
*Aeromonas* (*n* = 1)	0.7
*Citrobacter freundii* (*n* = 4)	0.33
*Citrobacter koseri* (*n* = 2)	0.53
*E. coli* (*n* = 13)	0.55
*Enterobacter aerogenes* (*n* = 1)	0.3
*Klebsiella pneumoniae* (*n* = 16)	0.48
*Klebsiella oxytoca* (*n* = 4)	0.56
*Pseudomonas* (*n* = 4)	0.5
*Proteus vulgaris* (*n* = 3)	0.65
*Proteus mirabilis* (*n* = 1)	0.65

**Table 2:** MAR index value of sampling site.

Sampling Sites (*n* = Isolate)	MAR Index
SWW (*n* = 12)	0.51
HWW-01 (*n* = 12)	0.54
HWW-02 (*n* = 9)	0.53
HWW-03 (*n* = 16)	0.48

**Table 3:** ARI index for Gram-negative bacteria.

Organism Identified	Class of Antibiotics								
	Aminoglycoside	β-Lactam	Cephalosporin	Phenicols	Fluoroquinolones	Sulfonamides	Carbapenem	Quinolone	Tetracycline
*Citrobacter* (*n* = 6)	0.02	0.11	0.09	3.33	13.33	0	0.03	10	10
*Ecoli* (*n* = 13)	0.04	0.14	0.16	0.003	0.05	0.02	0.04	0.04	0.03
*Pseudomonas aeruginosa* (*n* = 4)	0	0.1	0.13	0.03	0.01	0.03	0.03	0.05	0.04
*Enterobacter aerogens* (*n* = 1)	0	0.05	0.05	0	0.05	0	0.05	0	0.05
*Aeromonas* (*n* = 1)	0	0.2	0.2	0.05	0.05	0	0	0.05	0.05
*Klebsiella* spp. (*n* = 20)	0.04	0.145	0.145	0	0.04	0.01	0.05	0.02	0.015
*Proteus* (*n* = 4)	0.03	0.13	0.16	0.05	0.04	0.05	0.04	0.05	0.05
*Citrobacter* (*n* = 6)	0.03	0.11	0.09	3.33	13.33	0	0.03	10	10

**Table 4:** ARI index for sampling sites.

Site	Class of Antibiotics								
	Aminoglycoside	β-Lactam	Cephalosporin	Phenicols	Fluoroquinolones	Sulfonamides	Carbapenem	Quinolone	Tetracycline
SWW (*n* = 12)	0.03	0.15	0.14	0.004	0.04	0.01	0.04	0.03	0.017
HWW-01 (*n* = 12)	0.05	0.12	0.13	0.01	0.03	0.02	0.05	0.03	0.03
HWW-02 (*n* = 9)	0.03	0.12	0.15	0.01	0.03	0.02	0.04	0.02	0.03
HWW-03 (*n* = 16)	0.03	0.12	0.125	0.009	0.05	0.01	0.03	0.03	0.03

**Table 5:** Oligonucleotides used for the detection of antibiotic resistance genes in Gram-negative bacteria.

Antibiotic	Primer	Primer Sequence (5′-3′)	Size (bp)	Reference
Penicillin and cephalosporins	*bla*_SHV_	F-AAAGCGAAAGCCAGCTGTCG	656	[40]
		R-GTTATTCGGGCCAAGCAGGG		
Ampicillin	*bla*_TEM_	F-CGCCCCGAAGAACGTTTTCC	329	
		R-CGTTGGGAACCGGAGCTG		
Cefotaxime	*bla*_CTX-M_	F-CGGTGCAACAAAAGCTGGCG	503	
		R-GCGGCTGGGTAAAATAGGTC		
CTX-M group I	*bla*_CTM-M15_	F-ATCACTGCGCCAGTTCACGCT	584	[41]
		R-GGCTGGGTGAAGTAAGTGACC		
Carbapenem	*bla*_NDM-1_	F-GGTTTGGCGATCTGGTTTTC	621	[42]
		R-CGGAATGGCTCATCACGATC		
	*bla*_IMP_	F-GGAATAGAGTGGCTTAACTCTC	232	
		R-GGTTTAACAAAACAACCACC		
	*bla*_VIM_	F-GATGGTGTTTGGTCGCATA	390	
		R-CGAATGCGCAGCACCAG		
	*bla*_KPC_	F-CGTCTAGTTCTGCTGTCTTG	798	
		R-CTTGTCATCCTTGTTAGGCG		
	*bla*_OXA-48_	F-GCGTGGTTAAGGATGAACAC	438	
		R-CATCAAGTTCAACCCAACCG		
Tetracycline	*tet*D	F-GCAAACCATTACGGCATTCT	546	[43]
		R-GATAAGCTGCGCGGTAAAAA		
	*tet*E	F-TATTAACGGGCTGGCATTTC	544	
		R-AGCTGTCAGGTGGGTCAAAC		
	*tet*G	F-GCTCGGTGGTATCTCTGCTC	550	
		R-CAAAGCCCCTTGCTTGTTAC		
Chloramphenicol	*cat*A1	F-AACCAGACCGTTCAGCTGGAT	549	
		R-CCTGCCACTCATCGCAGTAC		
	*cat*A2	F-AACGGCATGATGAACCTGAA	547	
		R-ATCCCAATGGCATCGTAAAG		
Sulfonamide	*sul*III	F-ATGAGCAAGATTTTTGGAATCGT	792	
		R-CTAACCTAGGGCTTTGGATATTT		
Plasmid-mediated quinolone resistance genes Quinolones	*qnr*A	F-ATTTCTCACGCCAGGATTTG	516	[44]
		R-GATCGGCAAAGGTTAGGTCA		
	*qnr*B	F-GATCGTGAAAGCCAGAAAGG	469	
		R-ACGATGCCTGGTAGTTGTCC		
	*qnr*S	F-ACGACATTCGTCAACTGCAA	417	
		R-TAAATTGGCACCCTGTAGGC		
Fluoroquinolone	*qepa*	F-CGTGTTGCTGGAGTTCTTC	403	
		R-CTGCAGGTACTGCGTCATG		

## Data Availability

The data are contained within the article.

## Supplementary Materials

The following supporting information can be downloaded at: https://www.mdpi.com/article/10.3390/antibiotics14060607/s1, Table S1: Distribution of AR-GNB strains isolated from sampling sites.

## Abbreviations

The following abbreviations are used in this manuscript:

AMR	Antimicrobial resistance
AR	Antibiotic resistance
ARB	antibiotic-resistant bacteria
AR-GNB	Antibiotic-resistant Gram-negative bacteria
ARGs	antibiotic resistance genes
ARI	Antibacterial resistance index
EMB	Eosin Methylene Blue
HWW	Hospital wastewater
JPIAMR	The Joint Programme Initiative on Antimicrobial Resistance
MAR	Multiple antibiotic resistance
MDR	Multidrug resistance
PDR	Pan-drug-resistant
QRDRs	Quinolone resistance-determining regions
TCBS	Thiosulfate Citrate Bile sucrose
TSA	Trypticase Soya Agar
WHO	World Health Organization
XDR	extensive drug resistance
WWTPs	Wastewater Treatment Plants
