# Supplementary material for: Surveillance of Multidrug-Resistant Genes in Clinically Significant Gram-Negative Bacteria Isolated from Hospital Wastewater
Source: Antibiotics (Basel). 2025 Jun 15;14(6):607. doi: 10.3390/antibiotics14060607 (PMC12189587; doi:10.3390/antibiotics14060607)
Supplement: Supplementary file 1 [file antibiotics-14-00607-s001.zip › antibiotics-3608001-supplementary.pdf]

Table S1. Distribution of AR-GNB strains isolated from sampling sites.

Sampling sites (*n*= isolate)	AR-GNB isolated
SWW (*n*=12)	*Citrobacter freundii* (*n*=1), *E. coli* (*n*=3), *Klebsiella pneumoniae* (*n*=6),*Klebsiella oxytoca* (*n*=1), *Pseudomonas aeruginosa* (*n*=1)
HWW-01 (*n*=12)	*Citrobacter freundii* (*n*=1), *E. coli* (*n*=3), *Klebsiella pneumoniae* (*n*=3), *Klebsiella oxytoca* (*n*=2), *Pseudomonas aeruginosa* (*n*=1), *Proteus vulgaris* (*n*=2)
HWW-02 (*n*=9)	*Citrobacter freundii* (*n*=1), *E. coli* (*n*=3), *Enterobacter aerogenes* (*n*=1),*Klebsiella pneumoniae* (*n*=2), *Pseudomonas aeruginosa* (*n*=1), *Proteus mirabilis* (*n*=1)
HWW-03 (*n*=16)	*Aeromonas spp*. (*n*=1), *Citrobacter freundii* (*n*=1), *Citrobacter koseri* (*n*=2), *E. coli* (*n*=4), *Klebsiella pneumoniae* (*n*=5), *Klebsiella oxytoca* (*n*=1), *Pseudomonas aeruginosa* (*n*=1),*Proteus vulgaris* (*n*=1)
